# Fine needle aspiration cytology of bone tumours- the experience from the National Orthopaedic and Lagos University Teaching Hospitals, Lagos, Nigeria

**DOI:** 10.1186/1742-6413-3-16

**Published:** 2006-06-15

**Authors:** Obiageli E Nnodu, SO Giwa, Samuel U Eyesan, Fatima B Abdulkareem

**Affiliations:** 1 Departments of Pathology & Orthopaedic and Trauma Surgery, National Orthopaedic Hospital Lagos, Nigeria; 2 Orthopaedic Unit, Department of Surgery, Lagos University Teaching Hospital, Lagos, Nigeria; 3 Departments of Pathology, National Orthopaedic Hospital Lagos & Department of Morbid Anatomy, Lagos University Teaching Hospital, Lagos, Nigeria

## Abstract

**Background:**

Due to difficulty in confirming clinical suspicions of malignancy in patients presenting with bone tumours, the cost of surgical biopsies where hospital charges are borne almost entirely by patients, competition with bone setters and healing homes with high rate of loss to follow up; we set out to find if sufficient material could be obtained to arrive at reliable tissue diagnosis in patients with clinical and radiological evidence of bone tumours in our hospitals.

**Methods:**

After initial clinical and plain radiographic examinations, patients were sent for fine needle aspirations. Aspirations were carried out with size 23G needles of varying lengths with 10 ml syringes in a syringe holder (CAMECO, Sebre Medical, Vellinge, Sweden). The aspirates were air dried, stained by the MGG method and examined microscopically. Histology was performed on patients who had subsequent surgical biopsy. These were then correlated with the cytology reports.

**Results:**

Out of 96 patients evaluated, [57 males, 39 females, Mean age 31.52 years, Age Range 4–76 years,] material sufficient for diagnosis was obtained in 90 patients. Cytological diagnosis of benign lesions was made in 40 patients and malignant in 47. Of these, 27 were metastases, osteogenic sarcoma 16, giant cell tumour 19, infection 11. Histology was obtained in 41 patients. Correct diagnosis of benignity was made in 17 out of 18 cases, malignancy in 21 out of 22 cases. One non-diagnostic case was malignant. The accuracy of specific cytological diagnosis was 36/41 (87.8%) and incorrect in 5/41 (12.2%).

**Conclusion:**

We conclude that FNAC can be useful in the pre-operative assessment of bone tumours especially where other diagnostic modalities are unavailable.

## Background

The spectrums of diagnostic options for bone tumours in our environment are largely unavailable to majority of patients. Surgical biopsies are not always possible in patients suspected to have bone tumours on clinical and radiological grounds due to cost, competition with bone setters and healing homes and high rate of loss to follow up. When biopsies are performed, there is usually a long waiting time in processing bone specimens.

As reported in literature, although most bone tumours can be diagnosed on the basis of plain X-rays, [1] biopsy is an important aspect of the preliminary investigation. Open biopsy is the considered procedure of choice for diagnostic tissue sampling but it requires hospitalization, sometimes contaminates surrounding tissues, with an associated risk of infection, haematoma formation and pathological fracture [2].

Fine Needle Aspiration cytology is a quick, safe, cheap and reliable diagnostic tool for the evaluation of masses from different sites in the body but it has not been widely applied in the diagnosis of bone tumours. This could be due to technical problems, the morphological heterogeneity of bone tumours and anticipated difficulty in obtaining adequate tissue material or mainly due to limited experience since tumours of the musculoskeletal system are rare [1,2].

In recent times however, in specialized centres abroad, core needle biopsy and Fine Needle Aspiration are gradually becoming more accepted as a substitute for traditional open biopsy. FNA can be safely performed in difficult sites such as the vertebrae or pelvis, does not require hospitalization, allows preliminary diagnosis within 15–20 minutes of aspiration and adjunctive methods such as electron microscopy, immunocytochemistry, cytogenetics and DNA-ploidy studies can be employed to arrive at a definitive diagnosis [3].

We report on our experience on the use of Fine Needle Aspiration Cytology in the diagnosis of bone tumours in our hospitals. This initiative was driven by concern about the high rate of loss to follow up in patients who are suspected on clinical and radiological grounds to have bone tumours. Very often, the patients are unable to go for surgery and tissue diagnosis cannot be obtained.

To the best of our knowledge, this is the first report of such a study in bone tumours in the country.

## Methods

The subjects were patients who presented with bone tumours at the National Orthopaedic Hospital, Igbobi, Lagos and the Orthopaedic Unit of the Department of Surgery, Lagos University Teaching Hospital between May 1997 and October 2002.

After full clinical examination, they were sent with X-rays of the affected bone for fine needle aspiration cytology. The aspirations were carried out using size 23G needles of varying lengths with 10 ml syringes in a syringe holder (CAMECO, Sebre Medical, Vellinge, Sweden) to make 2 or more passes in different aspects of the lesion as identified on X-ray and clinical examination. In two patients, this procedure had to be carried out under image intensifier. The aspirates obtained were spread on frosted-ended glass slides, air-dried, stained by the May Grunwald Giemsa technique and examined microscopically. Ten patients who had intra cortical lesions, which could not be reached with the use of fine needles, were excluded from the study.

## Results

Out of 96 patients [57 males, 39 females, mean age 31.52, age range 4–76 years] seen between May 1997 and October 2002, only 41 (42.7%) had subsequent surgical biopsy and histology. Histology/cytology correlation could only thus be carried out on this number. Material sufficient for diagnosis was obtained in 90 patients. In 6 patients, the material obtained was not enough for analysis. Cytological diagnosis of benign lesions was made in 40 patients and malignant in 47 patients. Of these 27 were metastases (28.1%) osteogenic sarcoma 16 (16.6%), giant cell tumour 19, (19.8%) infection 11, (11.5%), etc (Table 1).

**Table 1:** Cytological diagnosis of bone lesions.

**Metastasis:**	
Plasma cell lesion	8
Sarcoma	3
Metastatic carcinoma	16 = 27
Giant cell tumour	19
Osteosarcoma	16
Infection	11
Chondroid tumour	4
Fibrous dysplasia	2
Fibrohistiocytic tumour	3
Osteoblastoma	2
Bone cyst	2
Reactive	1
No cytological diagnosis	3
Insufficient for diagnosis	6

**TOTAL**	**96**

### Diagnostic accuracy

Table 2 shows the overall diagnostic accuracy according to benign or malignant.

**Table 2:** FNA of 41 bone tumours: overall diagnostic accuracy.

FNAC	Histology	
FNA Diagnosis	Confirmed as: Benign	Malignant

Benign n = 18	17	1
Malignant n = 22	1	21
Non diagnostic aspirate n = 1	-	1

In the 41 patients with available histology (Table 3), 36 patients had the correct cytological diagnosis (87.8%) and in 5 patients (12.2%) there was an incorrect specific diagnosis but when considered in terms of benign or malignant cytological diagnosis correlated with histology, the sensitivity was 95% and specificity, 94%.

**Table 3:** Accuracy of specific cytological diagnosis of bone tumours in 41 patients with available histology

Cytological Diagnosis	No of cases	Correct	Incorrect
Plasma cell neoplasm	4	4	-
Metastatic carcinoma	5	4	1(Rhabdomyosarcoma)
Giant cell tumour	10	10	-
Osteosarcoma	11	10	1(Fibrous dysplasia)
Infection	5	4	1 (Malignant fibrohistiocytic tumour)
Chondroid tumour	1	1	-
Fibrous dysplasia	1	1	-
Osteoblastoma	1	1	1
Firohistiocytic tumour	-	1	1 (Haemangioendotheliosarcoma)
Reactive	1	1	-
Non diagnostic aspirate	1	-	1 (Osteosarcoma)

**Total**	41	36 **(87.8%)**	5 **(12.2%)**

### Details on discordant cases

In one patient, a diagnosis of malignant fibrohistiocytic tumour was made but the surgical biopsy came out as haemangioendotheliosarcoma. Cytology correctly identified this as malignant but not the type. One fibrous dysplasia was wrongly diagnosed as osteosarcoma. The use of alkaline phosphatase technique would have shown that the atypical cells seen in the fine needle aspiration were not producing osteoid but this was not available to us. In the third patient, a 17 year old male with a mass in the right scapula and axillary region, an intense inflammatory infiltrate was noted with neutrophils, eosinophils, lymphocytes and three dimension balls of small cells, some large pleomorphic hyperchromatic naked nuclei, smudge cells, mesh work of fibres in some areas and spindle cells. A differential diagnosis of (a) metastatic carcinoma; query primary site (b) Other small round cell tumours (e.g. neuroblastoma, rhabdomyosarcoma, Ewing's sarcoma) was made with a recommendation for open biopsy. A surgical biopsy and histology revealed a rhabdomyosarcoma. Small round cell tumours show a uniform light microscope picture [4] and the differential diagnosis is often resolved with the use of ancillary techniques that were unavailable to us.

In the fourth patient, a 17-year-old male presented with a (L) elbow mass which yielded 7 ml of straw coloured slightly gelatinous fluid on aspiration. Cytology showed only a sub acute inflammatory picture but since the clinical picture was malignant with progressive weight loss and weakening radial pulse, the tumour was resected and histology showed malignant fibrous histiocytoma. This was probably because the aspiration needles could only reach the inflammatory reactive zone surrounding the tumour.

In the last patient, there was a non-diagnostic aspirate with a recommendation for open biopsy, which turned out to be a parosteal osteosarcoma. This is not an unusual finding since this lesion is noted for having quite subtle cytological signs of malignancy [5].

### Anatomic sites of the tumours

Table 4 shows the distribution of tumours according to anatomic sites. The femur (n = 28) accounted for the highest followed by the Tibia (n = 19) Humerus (n = 18). The least were the vertebrae scapula and ribs with (n = 1) each.

**Table 4:** Distribution of tumours according to anatomic sites.

Site	Number
Vertebrae	1
Sacrum	2
Pelvis	5
Rib	1
Scapula	1
Clavicle	3
Humerus	18
Radius	6
Hand	4
Femur	28
Tibia	19
Fibula	5
Foot	5
Other	5

Total	103

For the 27 patients with metastatic tumours 8 were plasma cell lesions, sarcoma 5 and carcinoma 16. Available radiological investigation showed lytic bone lesions in 14 patients while 5 patients had evidence of pathological fractures. The commonest sites for the metastasis were femur (6) pelvis (5) Tibia (3), clavicle (3) etc whilst least common sites are mainly the flat bones (Table 5).

**Table 5:** Sites of bone metastasis

Femur	6
Pelvis	5
Humerus	4
Clavicle	3
Tibia	3
Mandible	1
Cranium	1
Scapula	1

## Discussion

FNA of bone has been especially successful in the diagnosis of malignant tumours. When the material is sufficient, it is usually possible to define the tumour type as well as diagnose malignancy. Differentiation between a primary and metastatic lesion can also be made by this method [6]. The cytological features of FNA bone tumours have been reported previously [[3,6] and [7]]. As many malignant primary bone tumours have palpable soft tissue extensions, which are easily aspirated, we used size 23G needles for most of the aspirations. The choice of the length to use depended on the size of the tumour and plain radiographs were useful to determine this appropriately. Needles of shorter lengths only hit the reactive zone and lead to inconclusive or erroneous results. Most patients presented late when the cortex was already breached but the unavailability of coaxial biopsy needles with CT guidance was a limitation, as intra cortical lesions could not be sampled.

In this study, air-dried smears for May Grunwald Giemsa stains were employed but aspirates can also be fixed immediately without air-drying in 96% Alcohol and stained by the Haematoxylin and Eosin method. This has the advantage of better nuclear detail and ease of comparison with histological sections, which is more familiar to pathologists.

Metastatic tumours (n = 27) accounted for 28.1% of bone tumours in this study, which is comparable to 26% reported by Kabukçuoglu [6]. In 6 cases, the primary sites were suggested by the presence of tumours in the breast (2 cases), thyroid (2 cases), and prostate (2 cases). None was confirmed by FNA as we did not have the facility for immunocytochemistry. No attempt was made in this study to sample lesions within vertebrae due to the absence of requisite radiological imaging modalities.

**Figure 1:**
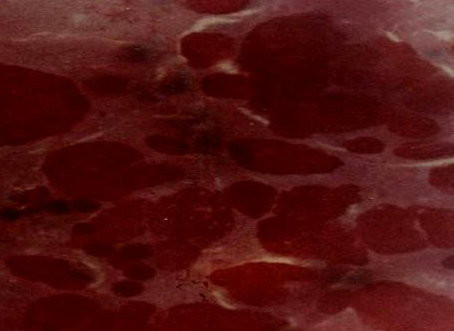
Osteosarcoma showing nuclear pleomorphism and a tumour giant cell.

As reported elsewhere osteosarcoma was the highest primary malignant tumour [5]. The clinical features of age at presentation, rapid onset, X-ray picture of irregularly outlined osseous defect with cortical destruction, sun burst appearance and Codman's triangle with the cytological features of single cells admixed with loose or cohesive clusters, hyperchromatic, pleomorphic nuclei, prominent nucleoli, large multinucleated tumour giant cells, mitotic figures and the presence of an osteoid matrix were essential to the diagnosis. (Fig. 1)

The importance of the aid of radiological features for FNA was underscored by one patient who was sent without an X-ray and the initial aspiration showed intense osteoblastic reaction with some atypical cells but as this was at variance with the clinical findings, a repeat aspirate using plain X-ray to properly localize the tumour contained characteristic malignant cells enabling the correct diagnosis to be made.

**Figure 2:**
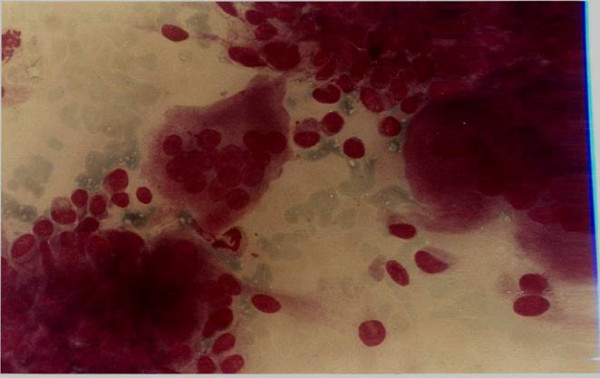
Giant cell tumour showing mononuclear single cells and large multinucleated osteoclast type giant cells attached to the periphery of clusters of spindle cells.

**Figure 3:**
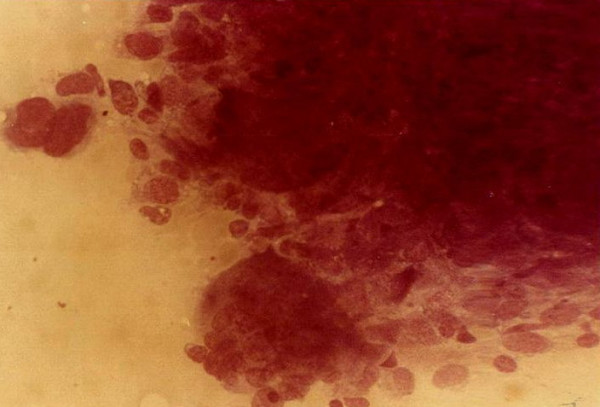
Giant cell tumour with marked cellular hyperplasia, increase in stromal cells with nuclear pleomorphism.

**Figure 4:**
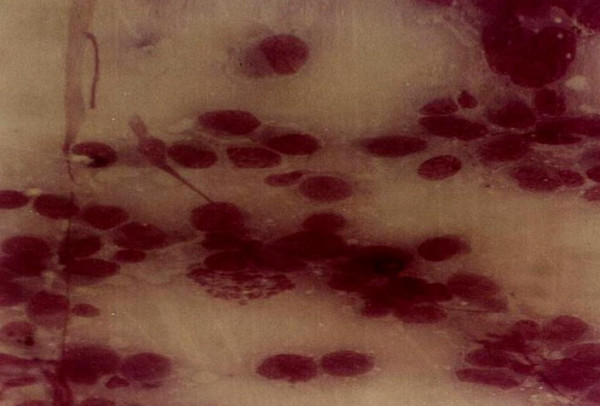
Same case as in fig 3 with features of malignancy and mitotic figures.

Giant cell tumour (Fig. 2) was the most common benign tumour of bone. The typical cytological picture is that of dispersed cells and cohesive clusters with mononuclear single oval to spindle cells and large multinucleated osteoclast type giant cells which are often attached to the periphery of the cluster of spindle cells [3]. The nuclei of the giant cells are bland. Again the clinical and radiological findings were very important as there are many lesions which contain numerous osteoclast type giant cells. In one patient a repeat FNA on a rapidly growing right shoulder tumour previously diagnosed clinically and radiologically and by FNA as giant cell tumour showed marked cellular hyperplasia with increase in stromal cells, marked nuclear features of malignancy and numerous mitotic figures (Figs 3 &4). An impression of suspicion for malignancy in an actively growing focus in giant cell tumour was made and this was confirmed later histiologically [9].

The differential diagnosis between osteomyelitis and neoplasm may be difficult clinically and radiologically, therefore the result of bacterial culture and the findings of mostly inflammatory cells helped to establish the diagnosis of osteomyelitis. The difficulties encountered were achieving the correct anatomical localization of the tumours, the utter reliance on morphologic criteria, poor record keeping, inability to access intra cortical lesions, failure to follow through with surgical biopsy in 57.3% of cases and the limited experience. The use of image guided FNA can help to localize deep-seated lesions appropriately and immunocytochemistry and other ancillary techniques can help to resolve morphologic deadlocks.

## Conclusion

We conclude that FNA can be employed with good clinical and plain radiological input to arrive at a preliminary diagnosis in patients with bone tumours where other diagnostic modalities are limited.

## Abbreviations

1. FNA- Fine needle aspiration.

2. FNAC- Fine needle aspiration cytology.

## Competing interests

The author(s) declare that they have no competing interests.

## Authors' contributions

SOG conceived the study, performed the surgical biopsies, and participated in the analysis of the data and preparation of the manuscript.

OEN carried out the fine needle aspirations, reported the cytology smears, analyzed the data and prepared the manuscript.

SUE Participated in the study design performed some surgical biopsies, data collation and reviewed the manuscript.

FBA carried out the histology reports and reviewed the manuscript.

All authors read and approved the manuscript.

**Figure 5:**
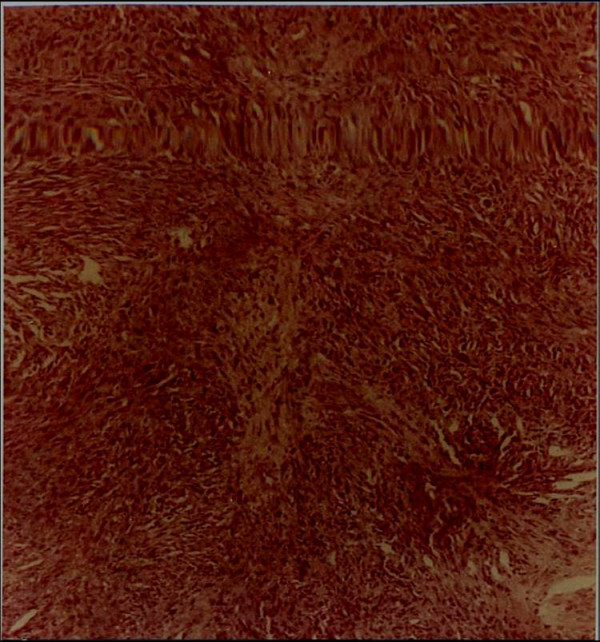
Histology of case in Figs 3 & 4.
